# Study of Viral Photoinactivation by UV-C Light and Photosensitizer Using a Pseudotyped Model

**DOI:** 10.3390/pharmaceutics14030683

**Published:** 2022-03-21

**Authors:** Mohammad Sadraeian, Fabio Francisco Pinto Junior, Marcela Miranda, Juliana Galinskas, Rafaela Sachetto Fernandes, Edgar Ferreira da Cruz, Libing Fu, Le Zhang, Ricardo Sobhie Diaz, Gustavo Cabral-Miranda, Francisco Eduardo Gontijo Guimarães

**Affiliations:** 1 Instituto de Física de São Carlos, Universidade de São Paulo, Caixa Postal 369, São Carlos 13560-970, SP, Brazil; fabiojr@ifsc.usp.br (F.F.P.J.); marcelamiranda@usp.br (M.M.); rafaela.fernandes@usp.br (R.S.F.); 2 Institute for Biomedical Materials and Devices (IBMD), Faculty of Science, University of Technology Sydney, Sydney, NSW 2007, Australia; libing.fu@uts.edu.au (L.F.); leo.zhang@uts.edu.au (L.Z.); 3 Laboratório de Retrovirologia, Escola Paulista de Medicina, Universidade Federal de São Paulo, São Paulo 04039-032, SP, Brazil; biomedicaju@hotmail.com (J.G.); edgar.cruz@unifesp.br (E.F.d.C.); rsdiaz@catg.com.br (R.S.D.); 4 Department of Immunology, Institute of Biomedical Sciences, University of São Paulo (ICB/USP), São Paulo 05508-000, SP, Brazil; gcabral.miranda@usp.br; 5 Institute of Research and Education in Child Health (PENSI), São Paulo 01228-200, SP, Brazil

**Keywords:** viral inactivation, photodynamic inactivation, SARS-CoV-2 pseudovirus, enveloped virus, UV-C light, photosensitizer

## Abstract

Different light-based strategies have been investigated to inactivate viruses. Herein, we developed an HIV-based pseudotyped model of SARS-CoV-2 (SC2) to study the mechanisms of virus inactivation by using two different strategies; photoinactivation (PI) by UV-C light and photodynamic inactivation (PDI) by Photodithazine photosensitizer (PDZ). We used two pseudoviral particles harboring the Luciferase-IRES-ZsGreen reporter gene with either a SC2 spike on the membrane or without a spike as a naked control pseudovirus. The mechanism of viral inactivation by UV-C and PDZ-based PDI were studied via biochemical characterizations and quantitative PCR on four levels; free-cell viral damage; viral cell entry; DNA integration; and expression of reporter genes. Both UV-C and PDZ treatments could destroy single stranded RNA (ssRNA) and the spike protein of the virus, with different ratios. However, the virus was still capable of binding and entering into the HEK 293T cells expressing angiotensin-converting enzyme 2 (ACE-2). A dose-dependent manner of UV-C irradiation mostly damages the ssRNA, while PDZ-based PDI mostly destroys the spike and viral membrane in concentration and dose-dependent manners. We observed that the cells infected by the virus and treated with either UV-C or PDZ-based PDI could not express the luciferase reporter gene, signifying the viral inactivation, despite the presence of RNA and DNA intact genes.

## 1. Introduction

Novel coronavirus disease (COVID-19), caused by the SC2 virus, was first detected in December 2019 in the Hubei province of China, and has since sparked a global health crisis, with 5.1 million deaths reported by the World Health Organization (WHO) as of November 20 in 2021 [1]. This pandemic situation demands urgent attention toward finding novel strategies that might contribute to the prevention of viral spread via the inactivation of virions on surfaces, aerosols, and the human body.

The UV-C light has been used in healthcare facilities for environmental disinfection (air, liquid, and solid surfaces) [2]. The efficacy of this inactivation may depend not only on the wavelength but also on factors such as the pathogens (e.g., bacterial or viral species), light output, and environmental conditions [3]. UV-C light at 254 nm radiation enables the deposit of the energetic photons during interaction with the coronavirus, damaging the viral genome, and, consequently, the virus replication and proliferation can theoretically be abrupted [4]. In the case of RNA viruses like SC2, UV irradiation forms several RNA photoproducts, primarily from adjacent pyrimidine nucleotides, such as uracil dimers, as well as RNA–protein cross-links [3]. The formation of the uracil dimer potentially leads to frameshift or point mutations in the genome, known as UV-signature mutations of virus [5]. Hence, we should remain vigilant about the long-term effects of irradiation-mediated strategies for viral inactivation. There are several studies on the effects of UV-C for LD90 viral inactivation based on the time and dose of irradiation [2,6,7]; however, the mechanism of action of how UV-C inactivates viruses is still unclear [7,8].

**Figure 1:**
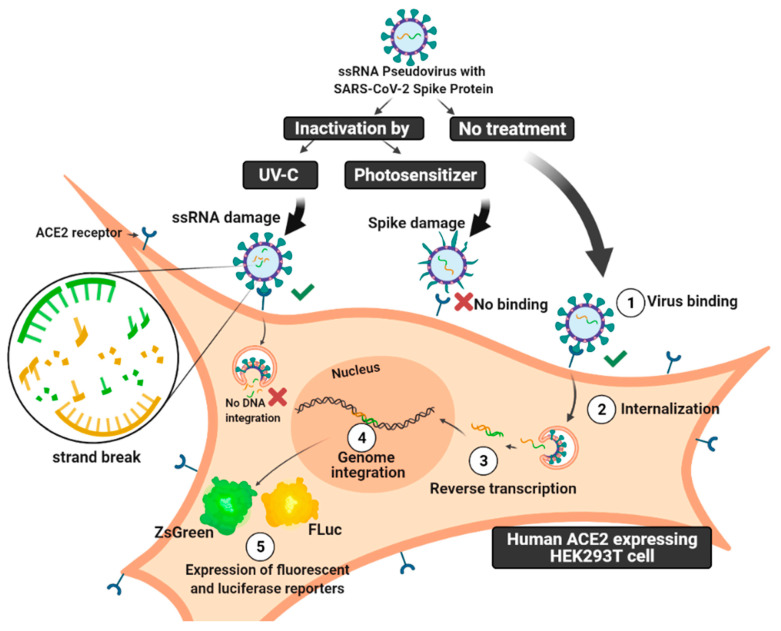
Schematic picture of the mechanism of SARS-CoV-2 pseudovirus infectivity. Unlike SC2 ssRNA virus, which has viral reproduction independent of the host genome, this counterpart pseudovirus carries on reporter ssRNA with LTR, which causes integration into the genome. In this study, the pseudovirus has been treated with either UV-C irradiation or photodynamic inactivation (PDI) by Photodithazine photosensitizer. The mechanism of infectivity of photo-inactivated pseudovirus particles has been compared on four levels; free-cell viral damage; viral cell entry; DNA integration; and expression of reporter genes. The figure was created with BioRender software.

Photodynamic therapy (PDT) is another light-based strategy that has been proposed to treat infections by damaging microorganisms, fungi, parasites, and viral particles. PDT is based on the use of photo-sensitive agents named photosensitizers (PS) which, in light-excited conditions and the presence of molecular oxygen, produce reactive oxygen species (ROS) [9,10,11,12,13,14,15]. PDT may damage cells via ROS generation, causing necrosis and apoptosis without harming the neighboring tissues. The advantages of utilizing photosensitizers for photodynamic inactivation (PDI) include its short-term toxicity, the absence of cell genome alterations, and avoiding the development of viral-induced resistance. Hence, the antiviral potential therapeutic effects of PDT and PDI on SC2 have been investigated with promising results [16,17]. Photoditazine photosensitizer (PDZ) is a porphyrin derivative with a chlorine core which allows it a high absorption in the red light spectrum with λmax of between 650–670 nm, as an advantage compared to the first generation of photosensitizers which are porphyrin core-based and which absorbs wavelengths too short for superior tissue penetration [18]. Understanding the mechanism of viral photoinactivation is important in finding and optimizing light-based strategies to battle viral infection. There are several reports on the mechanisms of viral photoinactivation with limited experiments on virion damage and viral propagation [2,19,20] due to the restriction of working with highly pathogenic viruses like HIV and SC2 viruses. Addressing these containment issues, the setting up of pseudotyped models in BSL2 labs can speed up studying the viral–cell mechanism and neutralizing assay towards in vivo studies [21,22]. Herein, we introduced the application of a pseudotyped model for studying the viral mechanism on four levels; virion damage; viral-cell entry; DNA integration; and expression of reporter genes. In this study, we followed the effects of UV-C irradiation and PDI on viral spike proteins and ssRNA in a HIV-based pseudotyped model of SC2 containing the Luciferase-IRES-ZsGreen reporter gene. Finally, we aimed to study the pseudovirus during cell internalization, genome integration, and reporter gene expression, after undergoing treatments by UV-C and PDZ photosensitizer under different concentrations and conditions (Figure 1).

## 2. Materials and Methods

### 2.1. Chemical Reagents

All reagents were purchased from Thermo Fisher Scientific (Waltham, MA, USA), unless otherwise stated.

### 2.2. Cells and Viruses

The HEK 293T cells expressing ACE-2 receptor were gifted from BEI Resources as catalog number NR-52511. ACE-2 enzyme is a critical receptor for virus entrance into the host cell. The HEK 293T cells were used as control cells for assays, and for pseudovirus generation. The cells were maintained at 37 °C in 5% CO_2_ in DMEM medium-high glucose (DMEM-HG) with 10% fetal bovine serum (Gibco Invitrogen, Grand Island, NY, USA). HEK 293T is a derivative human cell line isolated from human embryonic kidneys (HEK) and expresses a mutant version of the SV40 large T antigen.

### 2.3. Plasmids

Vector backbone pMD2.G and Vector backbone psPAX2 were gifts from Didier Trono (Addgene plasmid # 12259 and # 12260, respectively). The other plasmids were donated by BEI Resources and their sequences are available at (https://www.beiresources.org/ (accessed on 11 February 2022)) with the following catalog numbers [23]:

HDM-IDTSpike-fixK (BEI catalog number NR-52514): Plasmid expressing under a CMV promoter the Spike viral entry from SARS-CoV-2 strain Wuhan-Hu-1 (Genbank NC_045512);

pHAGE-CMV-Luc2-IRES-ZsGreen-W (BEI catalog number NR-52516): Lentiviral backbone plasmid that uses a CMV promoter to express luciferase followed by an IRES and ZsGreen;

HDM-Hgpm2 (BEI catalog number NR-52517): lentiviral helper plasmid expressing HIV Gag-Pol under a CMV promoter;

HDM-tat1b (NR-52518): Lentiviral helper plasmid expressing HIV Tat under a CMV promoter.

pRC-CMV-Rev1b (NR-52519): Lentiviral helper plasmid expressing HIV Rev under a CMV promoter.

### 2.4. UV-Vis Spectroscopy

UV-Vis spectroscopy was used to determine plasmid and protein concentrations by using Nanodrop 1000 UV-Visible spectrophotometer (Thermo Fisher Scientific, Waltham, MA, USA) [24].

### 2.5. Dynamic Light Scattering

Hydrodynamic radii, electrophoretic mobility, and polydispersity of SC2 Spike-pseudovirus were measured before and after photo inactivation. For UV-C inactivation and PDI inactivation, we followed the inactivation protocols as explained in Section 2.7 and Section 2.8. Then, samples with 70 μL volume at 1 mg/mL in UV-transparent 96-well plates were measured using a DLS Wyatt Möbius (Wyatt Technologies, Dernbach, Germany) with incident light at 532 nm, at an angle of 163.5°. Samples were equilibrated at 25 ± 0.1 °C for 600 s before the measurements, and this temperature was held constant throughout the experiments. All samples were measured in triplicate with 10 acquisitions and a 5 s acquisition time. The change in the cumulant-fitted hydrodynamic radius in nanometers was monitored during the storage period. Results were calculated using the Dynamics 7.1.7 software (Wyatt Technologies, Santa Barbara, CA, USA).

### 2.6. Generation of Pseudovirus with SARS-CoV-2 Spike and Naked Control

SC2 Spike pseudotyped lentiviruses were generated by transfecting 293T cells, adjusted with the protocol explained by Thermo Fisher Scientific (Waltham, MA, USA). Briefly, seed 293T cells to be 95–99% confluent at transfection. At 16–24 h after seeding, the cells were transfected with the plasmids required for lentiviral production by using Lipofectamine 3000 Reagent (Thermo Fisher Scientific, Waltham, MA, USA) following the manufacturer’s instructions and using the following plasmid with 1 mL total volume per well of a six-well plate. The 293T cells were transfected with a lentiviral backbone plasmid encoding Firefly luciferase and ZsGreen reporter proteins, a plasmid expressing SC2 Spike, and plasmids expressing HIV-1 *gag*, *pol*, and *tat* proteins, to assemble the membrane of viral particles. The same protocol was followed to generate naked control pseudovirus without adding the viral entry plasmid encoding SC2 Spike. At 8 h post-transfection, the packaging medium was removed and replaced. At 24 h post-transfection, the entire volume of cell supernatant was harvested and stored at 4 °C. Then, 1 mL of fresh medium was replaced. At 52 h post-transfection, the entire volume of the cell supernatant was harvested. The pseudovirus product was aliquoted in small volumes of 400 µL and stored at −80 °C prior to use and underwent a single freeze-thaw.

### 2.7. Viral Inactivation Using UV-C Irradiation

A total of 40 μL of pseudovirus were diluted in 60 μL of DMEM-HG without supplementation in each well of a 96-well plate, which were exposed to the UV-C lamp 254 nm (HNS G5, OSRAM Germicidal Puritec, Munich, Germany) placed 1 cm above the plate to allow a uniform irradiance over the plate (10 ± 2 mW/cm^2^). Light was delivered by 1, 6, and 36 s corresponding to doses of 10, 60, and 360 mJ/cm^2^, respectively. Controls were not submitted to irradiation. After irradiation, aliquots of 80 μL were placed into the plates containing the previously seeded 293T/ACE2 cells and incubated for 8 h at 37 °C with 5% CO_2_ for viral adsorption. Then, 120 μL of DMEM-HG medium containing 12% fetal bovine serum was added, and the plate was incubated for 48 h at 37 °C with 5% CO_2_. Afterward, the cells were placed into a lysis buffer solution to proceed with either Firefly luciferase assay or proviral DNA assay. Results were normalized in relation to controls for the calculation of viral inhibition rates of each sample.

### 2.8. Photosensitizer-Based Photodynamic Inactivation

A total of 40 μL of pseudovirus were diluted in 60 μL of DMEM-HG without supplementation in each well of a 96-well plate. The Photodithazine photosensitizer (PDZ) (Photodithazine^®^ Company, Moscow, Russia) with a serial dilution of 10, 50, and 250 µg/mL was added, and incubated in the dark at RT (22 °C) for 15 min, then were irradiated using a homemade LED device at 670 nm (red light). All irradiations were performed with an irradiance rate of 30 mW/cm^2^ in a time-dependent manner of 1, 10, and 20 min which equal the light doses of 1.8, 18, and 36 J/cm^2^, respectively. Afterwards, the treated ssRNA viruses were either harvested for the viral RNA load and DLS characterization or were incubated with the 293T/ACE-2 cells, as described in Section 2.7. After that, the cells were harvested for previral DNA load and luciferase activity measurement.

### 2.9. Quantification of Viral RNA and Proviral DNA

The total ssRNA pseudovirus, before and after irradiation, was extracted and purified using the RNeasy Lipid Tissue Mini Kit, according to the manufacturer’s (QIAGEN, Hilden, Germany) protocol.

The pseudovirus was treated with either 36 s UV-C irradiation or 10 min PDI in the presence of 10 µg/mL PDZ. The viral RNA load refers to the virus genome of free-cell pseudovirus, before and after treatment. Viral load measurement was carried out using one-step reverse transcriptase (RT) and real-time PCR in a single buffer system using the Abbott Real Time on the automated m2000, over the dynamic range of detection of 40 to 10,000,000 copies/mL (Abbott, IL, USA) [25]. The protocol was followed as described by Kumar et al. for the TaqMan One-Step RT and PCR Master Mix Reagents Kit (Thermo Fisher Scientific, Waltham, MA, USA) with primers and probes for long terminal repeat (LTR) region of 640 bp, with two identical regions located at both ends of the either proviral DNA or RNA viral of SC2 pseudovirus. Briefly, a volume of 5 µL RNA sample and 20 µL Master Mix were used for a one-step RT-qPCR reaction with 20 µM forward primer (5-GCCTCAATAAAGCTTGCCTTGA-3); 20 µM reverse primer (5-GGGCGCCACTGCTAGAGA-3); 10 µM Taqman probe (5-FAM-CCAGAGTCACACAACAGACGGGCACA-TAMRA-3); and an Applied Biosystems 7500 Fast Real-Time PCR system (Thermo Fisher Scientific, Waltham, MA, USA), as reported previously [25,26,27].

Quantification of Proviral DNA were completed with the TaqMan Real-Time PCR Assay. The cells were infected with the treated virus (Section 2.7 and Section 2.8). The cells were harvested three days after infection, centrifuged, and separated the pellets. The number of infected cells containing proviral DNA of pseudovirus was measured using qPCR. The quantification was executed based on the previously published protocol [27,28] for amplification of proviral DNA of pseudovirus (region LTR) with the primers described in Section 2.9.

### 2.10. Flow Cytometry

Direct fluorescence detections were applied using flow cytometry (Becton-Dickson Accuri C6, Mountain View, CA, USA) to analyze the expression of ZsGreen in the 293T/ACE-2 cells incubated with treated pseudovirus, as explained before (Section 2.7 and Section 2.8). After 48 h, the ZsGreen-positive cells were harvested, fixed by 2% paraformaldehyde (PFA), quantified by blue laser (20 mW) irradiation at 488 nm and analyzed in the channel FL1: 533/30. The acquired data were analyzed by Flow-Jo software version 7.5 (Tree Star Inc., Ashland, OR, USA).

### 2.11. Luciferase Assay

The infected cells which were harvested before (Section 2.7 and Section 2.8) were lysed with 20 µL of Luciferase Cell Culture Lysis Reagent (Promega, Madison, WI, USA), then mixed with 100 µL of Luciferase Assay Reagent (Promega, Madison, WI, USA), and the light emission was measured.

### 2.12. Titration of Pseudovirus

The pseudovirus particles were titrated using a method similar to SC2 pseudovirus generation. Virus titers were determined by measuring relative luciferase units (RLUs). The HEK293T cells expressing human ACE-2 (293T-ACE-2) were produced to test the correlation between ACE-2 expression and SC2 pseudovirus susceptibility. Particles were generated in two forms; with a SC2 spike and a negative control without a viral entry protein. Both pseudo-typed particles harbored a Luciferase-IRES-ZsGreen backbone. In a mirror plate, the percentage of cell viability was measured during the viral infection with a serial dilution of the virus starting at 50 μL pseudovirus in a total volume of 100 μL (0.5) for the spike pseudovirus. After 8 h of pseudovirus incubation, the media were replaced with 150 µL fresh media. After 48 h incubation, the wells containing 50 µL pseudovirus were studied for cell confluency. Afterwards, the titers of pseudotyped particles were quantified by a Luciferase assay expressed in RLU, to determine the number of transducing particles per mL.

### 2.13. Confocal Microscopy

One day before UV-C or PDI treatment, 2 × 104 cells per well of 293T/ACE-2 cells were seeded on a multiple-chamber slide (Nalge-Nunc International, Naperville, Ill, USA). The next day, cells were incubated with treated pseudovirus, as explained before (Section 2.7 and Section 2.8). After 48 h, the ZsGreen-positive cells were washed four times with PBS, fixed by 2% PFA. Images were obtained with an inverted LSM 780 multiphoton laser scanning confocal microscope (Zeiss, Jena, Germany), a 63 × 1.2 water immersion objective to couple with the bottom side of the cover slip, and the Zeiss LSM software was used to treat the images. The wavelength of Argon ion laser at 488 nm was used to excite the expressed ZsGreen protein compared to cell autofluorescence. The molecular localization of ZsGreen was analyzed for each image pixel in spectral and channel modes in the ranges 492–700 nm and 492–537 nm, respectively. The cells’ autofluorescence were analyzed from 585 to 692 nm.

Each pixel was associated with an emission spectrum which allowed the spatial separation of the expressed ZsGreen fluorescence (bright blue-greenish color) and the cell auto-fluorescence (yellow-orange color). Considering the spectral of the cell autofluorescence (maximum at 575 nm) is almost constant, the expression of the ZsGreen by the active pseudovirus internalization would be promptly signaled by the spectral superposition of the protein emission at around 515 nm.

### 2.14. Statistical Analyses

Statistical analyses were performed using the GraphPad Prism version 8.0 (GraphPad Software, San Diego, CA, USA). Data are shown as mean and SEM of the indicated number of replicate values. If no error bar appears present, the error bars are smaller than, and obscured by, the symbol. The method for statistical comparison used was unpaired two-tailed Student’s *t*-test, unless specifically indicated otherwise.

## 3. Results and Discussion

### 3.1. Generation of Pseudovirus with SARS-CoV-2 Spike and Naked Control

The spike-pseudotyped lentiviral particles were generated, which can infect 293T cells expressing the human ACE-2 receptor. In parallel, the naked control pseudovirus was generated, which harbors a backbone plasmid-encoding luciferase-IRES-ZsGreen reporter, but without the SC2 Spike on the membrane (Figure 1).

### 3.2. Titration of Pseudovirus

**Figure 2:**
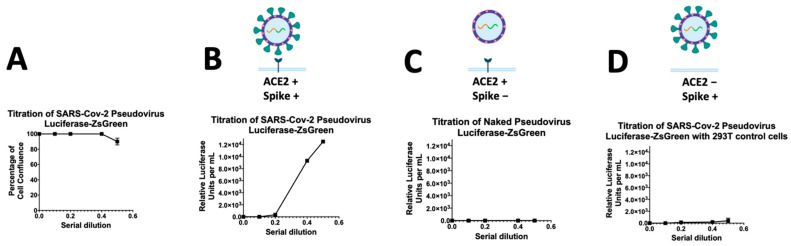
Titration of SARS-CoV-2 spike-pseudovirus particles in 293T cells expressing ACE-2. (**A**) Study of the percentage of cell viability during the viral infection respecting a serial dilution starting at 1:2 (0.5); (**B**,**C**) The graph shows the titers of the expression of Luciferase reporter as determined by measuring relative luciferase units (RLUs). The RLU data are the average of three-fold serial dilution of virus starting at 50 μL virus in a total volume of 100 μL (0.5) for the Spike-pseudovirus (**B**); naked pseudovirus without spike (**C**); or the Spike-pseudovirus with 293T cells without ACE2 receptor (**D**). After 8 h of pseudovirus incubation, the media were replaced with 150 µL fresh media.

The pseudovirus particles were titrated in two forms; particles with a SC2 spike and a negative control without a viral entry protein. Both pseudo-typed particles harbored a Luciferase-IRES-ZsGreen backbone. After 48 h incubation, the cell confluence reached 100% for all wells in a mirror plate containing non-transduced cells, however, the wells containing 50 µL pseudovirus showed 90% cell confluence (Figure 2A). The titers of pseudotyped particles were quantified by a Luciferase assay. Titers of >10^5^ RLUs per mL were measured in a 96-well plate (Figure 2B). Unsurprisingly, the ACE2-expressing cells incubated with naked pseudovirus without a spike did not show the expression of luciferase (Figure 2C). The other negative control was the incubation of 293T non-ACE2 control cells with Spike-pseudovirus, which did not show the luciferase expression, as expected (Figure 2D). In previous reports, researchers used polybrene to facilitate the lentiviral infection through minimizing charge-repulsion between the virus and cells [29], but we found this SC2 pseudovirus no need to polybrene for binding to the ACE-2 receptor.

### 3.3. Viral Inactivation Using UV-C Irradiation or Photosensitizer-Based PDI

**Figure 3:**
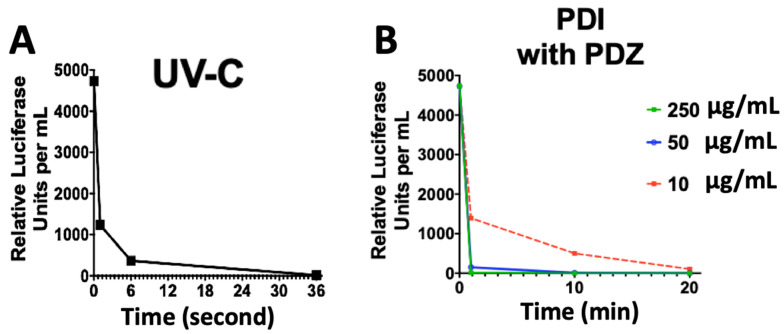
Study of the effect of photo-inactivation of ssRNA pseudovirus considering the relative luciferase units with a time-dependent manner of UV-C irradiation at 1, 6, and 36 s corresponding to doses of 10, 60, and 360 mJ/cm^2^, respectively (**A**); and PDZ-based PDI in a serial dilution of 10, 50 and 250 µg/mL in a time-dependent manner of 1, 10 and 20 min which equal the light doses of 1.8, 18 and 36 J/cm^2^, respectively (**B**). Data are ± means S.E.M. (*n* = 3).

A volume of 40 μL of pseudovirus was diluted in 60 μL of DMEM-HG without supplementation in each well of a 96-well plate, which were exposed to the UV-C lamp 254 nm for 1, 6, and 36 s corresponding to doses of 10, 60, and 360 mJ/cm^2^, respectively. Figure 3A represents the effect of UV-C irradiation on the photo-inactivation of ssRNA pseudovirus. The results showed that UV-irradiation may inactivate 74%, 93%, and 99.99% of SC2 Spike-pseudovirus particles during 1, 6, and 36 s irradiation, respectively. These results are comparable with the published results on SC2 elsewhere [20], due to the discrepancies of the cell-entry mechanism among virus and pseudovirus. Furthermore, a time-dependent manner of PDI was performed to find the maximum viral inactivation with the minimum time and concentration of Photogem PS (PDZ). As Figure 3B demonstrates, the viral inactivation depended on both time and the PS concentration. We observed that 99.8% of the pseudovirus were inactivated in the presence of 50 µg/mL PDZ with 10 min irradiation. Hence, we selected this time and concentration for further studies. These results indicate that both UV-C irradiation and PDI, as two distinct strategies, are highly effective in inactivating pseudovirus replication, while there could be some differences in the mechanism of infectivity between UV-C irradiation and PDI. Hence, we extended our studies focusing on the viral RNA and proviral DNA loads, as described in Section 3.4.

### 3.4. Study the Infectivity Mechanism of UV-C Irradiation and PDI Using qPCR

**Figure 4:**
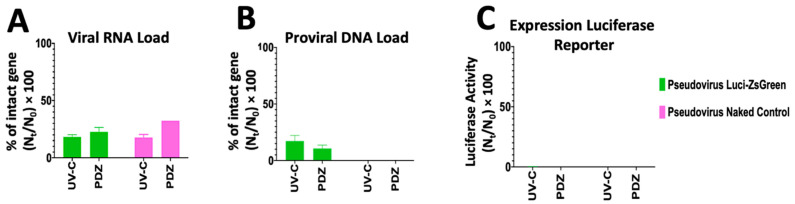
The pseudovirus has been treated with either 36 s UV-C irradiation or 10 min Photodynamic Inactivation in the presence of 50 µg/mL Photodithazine. The mechanism of infectivity of photo-inactivated pseudovirus particles has been compared on three levels; (**A**) viral RNA load referring to the free-cell virus; (**B**) proviral DNA load referring to the ability of the treated virus to complete the subsequent steps of cell internalization, reverse transcription and genome integration; (**C**) luciferase activity referring to the expression of luciferase reporter after DNA integration. n_t_/n_0_ represents the fraction of the targeted genome region that remained intact after treatment.

Viral inactivation could be due to either viral protein or viral genome damage [8,30,31]. We suppose that any damage to the virus spike may lead to loss of the virus binding ability and neutralization of the virus infectivity, while damaging the viral genome may affect the viral and proviral loads of pseudovirus. The results of the viral RNA load showed that both 36 s UV-C irradiation and PDZ-based PDI (10 min irradiation, 50 µg/mL PDZ) can damage ssRNA by 83% and 74%, respectively. The RNA of both control (naked pseudovirus without spike) and spike-positive viral particles were destroyed during irradiation (Figure 4A). By comparing the PDZ-based PDI in two forms of enveloped and non-enveloped (naked) viruses, we found out that PDI may damage the viral genome independently from the virus type.

The results of the proviral DNA assay may interpret the virus’s ability to complete the subsequent steps of cell binding, internalization, and genome integration after reverse transcription. The proviral DNA load of 36 s UV-C irradiation was as much as the RNA viral load, signifying that the UV-C based viral inactivation is independent of damaging the spike protein. In parallel, the proviral DNA load of PDZ-based PDI (10 min irradiation, 50 µg/mL PDZ) was decreased by 13%, which is half of the RNA viral load (26%), signifying the PDI-treated pseudovirus may lose cell infectivity due to damaging the spike (Figure 4A,B). Presumably, PDZ-based PDI destroys more of the spike than the viral genome, which leads to losing the binding ability of the virus. Unsurprisingly, the naked control particle showed no DNA load, as the control particle lacks a spike for cell binding. Furthermore, we observed that the cells infected by either UV-C or PDI-treated pseudovirus could not express the luciferase reporter gene (Figure 4C), signifying the total viral inactivation despite the presence of RNA and DNA intact genes.

In this study, n_t_/n_0_ represents the fraction of the targeted genome region that remained intact after treatment. In the viral RNA load, the targeted genome region is ssRNA of pseudovirus with LTR sequences. In the proviral DNA load, the targeted genome region is the integrated DNA of pseudovirus genome after reverse transcriptase. Unlike the SC2 virus, the mechanism of infectivity of the SC2 pseudovirus includes DNA integration, which is one of the advantages of utilizing the pseudotyped model. Therefore, we could follow a simple protocol for calculation of RNA and DNA load and compare the qPCR data with luciferase assay results, otherwise to estimate the infectivity based on qPCR data, the infectivity of virus should be assessed by estimation from the qPCR results, according to the protocol published by Sabino et al. [20,32].

### 3.5. DLS Measurements before and after Irradiation

**Figure 5:**
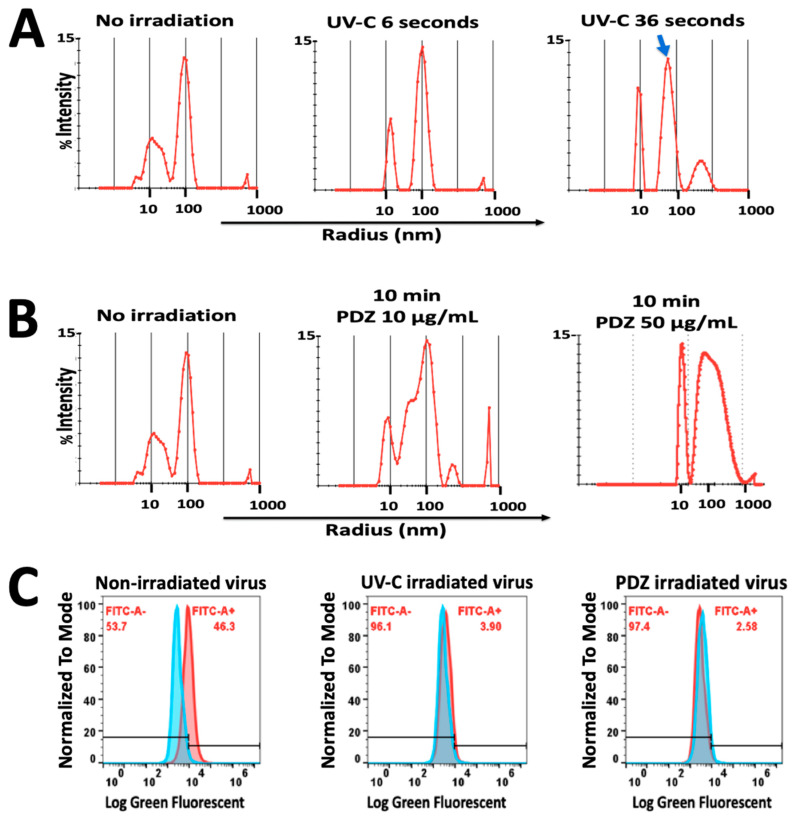
(**A**) Dynamic Light Scattering histograms of hydrodynamic radius (R_h_) for pseudovirus showed optimal polydispersity with R_h_ of 100 nm. During 36 s of UV-C irradiation (360 mJ/cm^2^), a slight decrease in the size of pseudovirus was observed (Blue arrow) with no significant aggregation; (**B**) PDZ-based PDI significantly affected the size and polydispersity of the virus, resulting in major aggregated particles in a higher concentration of PDZ (50 µg/mL); (**C**) Flow cytometric diagram on the left demonstrated the percentage of pseudovirus-infected cells expressing ZsGreen Fluorescent protein. The middle and right diagrams represent the cells infected by UV irradiated-virus and PDZ-based PDI virus, which do not express ZsGreen protein. FITC rate indicates the green fluorescent emission from ZsGreen. Data are ± means S.E.M. (*n* = 2).

DLS measurement demonstrated that 36 s UV-C irradiation on pseudovirus with 18 J/cm^2^ resulted in a slight decrease in the size distribution compared to the non-irradiated pseudovirus (Figure 5A). On the other hand, in the PDI study, the increase of PDZ concentration from 10 to 50 µg/mL had a significant effect on the size and polydispersity of the virus, and yielded significant aggregated particles (Figure 5B). We assumed that this aggregation may interrupt our results on the cell toxicity therefore we found that PDZ with 10 µg/mL was an appropriate concentration for further studies on flow cytometry and microscopy observations.

### 3.6. Green Fluorescent Measurement by Flow Cytometry

Furthermore, the cells were infected with viruses, which were treated with either 36 s of UV-C or PDZ (10 µg/mL) of PDI, to measure the expression of the ZsGreen protein. The flow cytometry results showed that 46.3% of the virus-infected cells were emitting green fluorescence, while the cells treated with UV-C or the PDI-treated viruses were not able to express the ZsGreen (Figure 5C).

### 3.7. Observation of ZsGreen Expression by Confocal Microscopy

**Figure 6:**
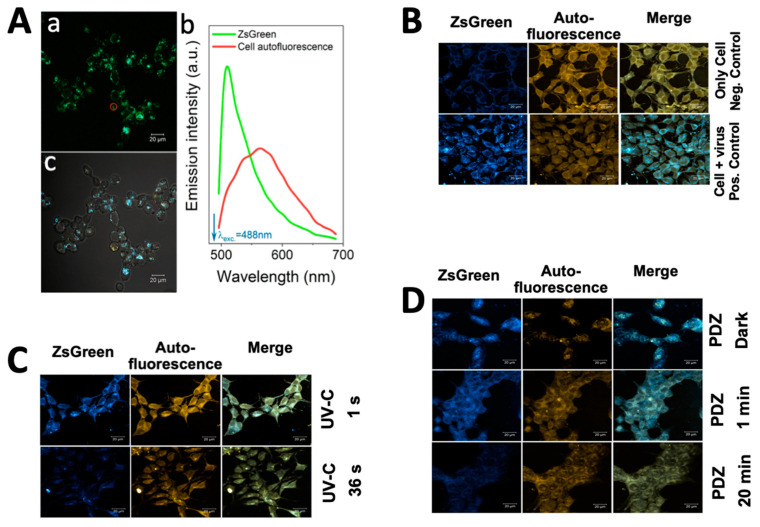
(**A**) Confocal microscopy images showing ZsGreen expression in 293T-ACE2 cells at 48 h after incubation with Spike-pseudotyped lentiviral particles with the ZsGreen backbone. The images are represented in spectral mode (panel (**a**)) and channel mode merged with a wide field transmission image (panel (**c**)). In panel (**b**), the green and red curves represent the regions of interest (ROI) of ZsGreen emission and the cell autofluorescence, respectively; (**B**) The positive control cells incubated with pseudovirus without treatment showed strong green fluorescent emission, indicating the expression of ZsGreen in comparison to negative control cells. The ZsGreen emission was detected between 492 and 532 nm (assigned the bright-blue false color), while the cellular autofluorescence can be differentiated by taking the emission (orange false color) in the spectral range from 585 to 695 nm; (**C**) The results of viruses with 36 s UV-C irradiation (360 mJ/cm^2^) did not show green fluorescent emission, while the cells with 1 s UV-C irradiation (10 mJ/cm^2^) were still showing somewhat ZsGreen expression; (**D**) The viruses were treated with Photoditazine (PDZ) (10 µg/mL) and irradiated in a time-dependent manner of 0 (so-called dark), 1 and 20 min. The cells infected by PDI virus after 20 min do not express ZsGreen protein. (**A**,**B**) scale bar: 20 µm.

Forty-eight h after cell incubation with pseudovirus (with no treatment), the ZsGreen expression was observed using confocal microscopy. Figure 6A shows images of the field in spectral mode (Figure 6A—panel (a)), and in channel mode merged with a wide field transmission image (Figure 6A—panel (c)). The two spectral contributions for both ZsGreen emission and the cell autofluorescence can be separated by taking two regions of interest (ROI) in panel (a) (green and red circles), as depicted as two graphs in Figure 6A—panel (b). In Figure 6A—panel (c) demonstrates that the emission detected between 492 and 532 nm (assigned the bright-blue false color) mainly signals the expression of the ZsGreen protein while the cellular autofluorescence can be differentiated by taking the emission (orange false color) in the spectral range from 585 to 695 nm.

To study the expression of ZsGreen protein by confocal microscopy, the cells were infected with pseudovirus treated with UV-C or PDI. The positive control cells incubated with pseudovirus without treatment showed strong green fluorescent emission indicating the expression of ZsGreen in comparison to negative control cells (Figure 6B). The results of viruses with 36 s UV-C irradiation (360 mJ/cm^2^) did not show green fluorescent emission (Figure 6C), while the cells with 1 s UV-C irradiation (10 mJ/cm^2^) were still showing slight ZsGreen expression. The results were in agreement with our luciferase assay (Figure 3A), confirming that the 1 s UV-C irradiation is not sufficient to completely inactivate the viruses.

For the PDI study, the viruses were incubated with 10 µg/mL PDZ, and irradiated for 1 or 20 min, which equals the light doses of 1.8 and 36 J/cm^2^, respectively (Figure 6D). The dark control groups were submitted to the same procedure, except for light exposure. No green fluorescent emission was observed in the cells after PDI with 20 min irradiation. In contrast, the dark controls showed fluorescent emission of ZsGreen. Neither PDZ irradiated samples nor dark samples showed toxicity on the cell confluency, while an increase of autofluorescence was observable, compared to the negative control cells. These observations confirm our results of luciferase assay (Figure 3B), and are in agreement with our previous studies on PDZ-based PDI, as described elsewhere [15].

In sum, two distinct strategies (UV-C irradiation and PDZ-based PDI) were applied for the inactivation of SC2 pseudovirus produced using HIV-based lentiviral system which specifically infect ACE2-expressing cells. This specificity was demonstrated using luciferase assay compared to the control negative cells and the control naked viruses, which agreed with previous reports [21,23,33,34]. The viral inactivation could be the consequence of either viral protein damage, which affects the cell internalization, or viral genome damage affecting the viral load. Unlike the SC2 RNA virus with viral reproduction independent of the host genome [7,8,30,35,36], this pseudotyped model enabled us to study not only the RNA viral load, but also the DNA integration, as well as the presence or absence of a spike on the viral particle. Several reports demonstrating the results of viral inactivation assays have a high degree of concordance with a clinical isolate of SC2 [33,34]; however, the results cannot be used for the inactivation of the actual SC2 virus unless tested.

## 4. Conclusions

Considering the advantages of pseudovirus over the actual SC2 virus, which was discussed above, we followed a simple protocol for calculating the RNA and DNA load and compared the qPCR data with luciferase assay results. Hence, we studied the viral inactivation by UV-C and PDI in dose and time-dependent manners via biochemical characterizations and quantitative PCR on four levels; virion damage; viral cell entry; DNA integration; and expression of reporter genes. Both UV-C and PDI treatments could destroy ssRNA and the spike protein of the virus in different ratios; however, the virus was still capable of binding and entering into the ACE-2 expressing 293T cells. UV-C irradiation disinfected the virus mainly through viral genome damage, with no apparent effects on the viral size and virus–cell binding ability. On the other side, PDZ-based PDI mostly destroyed the spike and viral membrane. Ignoring the type of viral destruction (ssRNA or spike), the cells infected by the photo-inactivated virus could not express the luciferase reporter gene. Our findings emphasize the advantages of PDI over UV-C viral inactivation. ROS-mediated damages on the viral envelope may generate debris or the fragments which could stimulate host immune defense. Moreover, viral PDI has affordability compared to other therapeutics like monoclonal antibodies (e.g., Ronapreve), which can be important factors for preventative use at home [37]. Other advantages of PDI include high repeatability without viral resistance or UV-signature mutations, with fast removal of the virus in a very short time.

The other advantage of this model is comparing the viral particles in two forms of enveloped and non-enveloped (naked) viruses, as a matter of importance for side-by-side comparison. Therefore, comparing two viruses with similar genomes but different in their protein envelope enables us to study the effect of each inactivation strategy on damaging the RNA genome in the presence and absence of a spike. Besides, this pseudotyped model can be used for other radiation-based strategies for understanding their mechanism of viral inactivation, with no need to work in BSL-3.

## Data Availability

All data available are reported in the article.

## Abbreviations

ACE-2	Angiotensin Converting Enzyme 2
DLS	Dynamic Light Scattering
PDI	Photodynamic Inactivation
PDT	Photodynamic Therapy
PDZ	Photoditazine
PS	Photosensitizer
RLU	Relative Luciferase Unit
SC2	SARS-CoV-2
